# Variations in *CCL3L *gene cluster sequence and non-specific gene copy numbers

**DOI:** 10.1186/1756-0500-3-74

**Published:** 2010-03-16

**Authors:** Sadeep Shrestha, Mawuli Nyaku, Jeffrey C Edberg

**Affiliations:** 1 Department of Epidemiology, University of Alabama at Birmingham, School of Public Health, 1665 University Blvd, RPHB Room 217L, Birmingham, Alabama 35294-0022, USA; 2 Division of Clinical Immunology and Rheumatology, University of Alabama at Birmingham, 1530 3rd Avenue South, Shelby 207, Birmingham, Alabama 35294-2182, USA

## Abstract

**Background:**

Copy number variations (CNVs) of the gene CC chemokine ligand 3-like1 (*CCL3L1*) have been implicated in HIV-1 susceptibility, but the association has been inconsistent. *CCL3L1 *shares homology with a cluster of genes localized to chromosome 17q12, namely *CCL3*, *CCL3L2*, and, *CCL3L3*. These genes are involved in host defense and inflammatory processes. Several CNV assays have been developed for the *CCL3L1 *gene.

**Findings:**

Through pairwise and multiple alignments of these genes, we have shown that the homology between these genes ranges from 50% to 99% in complete gene sequences and from 70-100% in the exonic regions, with *CCL3L1 *and *CCL3L3 *being identical. By use of MEGA 4 and BioEdit, we aligned sense primers, anti-sense primers, and probes used in several previously described assays against pre-multiple alignments of all four chemokine genes. Each set of probes and primers aligned and matched with overlapping sequences in at least two of the four genes, indicating that previously utilized RT-PCR based CNV assays are not specific for only *CCL3L1*. The four available assays measured median copies of 2 and 3-4 in European and African American, respectively. The concordance between the assays ranged from 0.44-0.83 suggesting individual discordant calls and inconsistencies with the assays from the expected gene coverage from the known sequence.

**Conclusions:**

This indicates that some of the inconsistencies in the association studies could be due to assays that provide heterogenous results. Sequence information to determine CNV of the three genes separately would allow to test whether their association with the pathogenesis of a human disease or phenotype is affected by an individual gene or by a combination of these genes.

## Background

A cluster of chemokines including the *CCL3L*-related gene family have been localized on chromosome 17q12 [1]. By sequencing a BAC clone located in this segmental duplication region, Modi identified 2 complete copies (*CCL3L1 *and *CCL3L3*) and one truncated copy (*CCL3L2*) of the *CCL3 *gene family [2]. It has been reported that individuals with more copies of *CCL3L1 *than their population median are less susceptible to HIV infection [3,4]. There seems to be a relationship between the copy number of *CCL3L1*-containing segmental duplications and viral load and HIV-specific CD4+ and CD8+ T cell responses [5]. The copy number of *CCL3L1 *influences risk of systemic lupus erythematosis (SLE) and modifies the SLE-influencing effects associated with the *CCR5 *genotype [6]; a higher copy number (> 2) of *CCL3L1 *is a risk factor for rheumatoid arthritis [7]. There is *CCR5-CCL3L1 *gene-gene interaction in susceptibility to Kawasaki disease [8], and higher numbers of infant *CCL3L1 *gene copies are associated with reduced HIV transmission [9,10]. In theory, the higher the copy number, the higher the ligand concentration, which should protect from HIV infection or disease progression. Chimpanzees, with higher copies (median of nine copy numbers), do not develop AIDS; this suggests biological significance. Copy number variation (CNV) of *CCL3L- *genes also affects the rate of progression to AIDS in rhesus macaques [11]. Other studies, however, do not show an association [12,13]. Although differences may exist in HIV progression outcomes, treatment variations, or interactions with the co-receptor CCR5 that result in differential associations, the methods used to determine CNVs have not been compared.

To avoid labor-intensive Southern blotting, the requirement for relatively large amounts of high-quality genomic DNA, and the inherent inaccuracies and high costs of other quantification techniques, high-throughput, real-time quantitative PCR (RT-PCR) assays have been developed to determine copy numbers. These procedures are applicable for *CCL3L1*. Since this gene cluster region is complex, some of the variations in the findings relevant to associations could be due to the different primers and probes used for the RT-PCR assays. To determine if this is the case, we evaluated different assays used to measure gene copy numbers of *CCL3L1*.

## Methods

We searched PubMed with the terms "MIP-1αP or MIP-1alpha P or LD78β or LD78beta or CCL3L1" for articles published in English up to the end of Jan 2010, and screened for publications specific to HIV/AIDS (and also separately for other diseases) and involving use of RT-PCR-based assays for examining copy numbers of *CCL3L1 *gene. Sequences of primers and probes used in RT-PCR assays in these publications were subjected to a nucleotide blast search against the human genomic plus transcript database on the National Center for Biotechnology Information (NCBI) website http://blast.ncbi.nlm.nih.gov/Blast.cgi. The sequences of genes containing these blast results were obtained from the UCSC Genomic Browser and aligned to the human reference sequence. Locations of exons, introns and untranslated regions of these genes were annotated from the NCBI Genome Browser. Pairwise, and multiple alignments were performed on these gene sequences via ClustalW in Molecular Evolutionary Genetics Analysis (MEGA 4) [14] and in BioEdit [15]. Primers and probes were aligned individually with each gene and then with all pre-aligned genes in MEGA 4 and BioEdit.

We used four different assays from the current literature (CCL3L_PP1, CCL3L_PP2, CCL3L_PP3, and CCL3L_PP4 in Table 1) in a previously described subset of 47 European American and 48 African American healthy controls [16] to quantify and examine the distribution of the copy numbers and the concordance rates between different assays in the two populations were assessed. Briefly, we used the specific primer/probe combinations (as shown in Table 1) to quantify the copy numbers of *CCL3L1 *with the single-copy gene hemoglobin, beta (*HBB*) serving as the internal control. Real-time PCR was performed using an AB 7500 Fast System (Applied Biosystems Inc.). Cycling conditions were: 2 min at 50°C, 10 min at 95°C, and 40 cycles of 15 s at 92°C and 1 min at 60°C. Test genomic DNA samples was diluted to obtain a concentration of 2.5 ng/μl, and 2 ul (5 ng genomic DNA) was used in each reaction. We ran each sample in triplicates across three 96-well plates. We used the Applied Biosystems relative quantification program to determine gene copy numbers for the individual samples. The *HBB *gene that is present at two copies per diploid genome was used to standardize the gene copy number counts. Consistent with previous studies, we performed RT-PCR for each individual in triplicate and determined the normalized relative copy number by generating a standard curve and then normalizing across samples by the results of the HBB control gene and dividing the value obtained by the reference individual. Rounding off the outcome from the previous step to the nearest integer provided the estimated copy number counts. The copy numbers matching in triplicates and/or duplicates was used as the final copy number for an individual sample.

**Table 1:** Specificity of primer and probes sets used in different HIV/AIDS and other disease association studies of *CCL3L*-related CNVs and mRNA expression.

#	Primer/Probe Sequences 5'-3' Sequence*	References		Specificity
		HIV/AIDS	Other diseases	
CCL3L_PP1	F: TCTCCACAGCTTCCTAACCAAGA	[3,4],	[6]^a^	*CCL3L1*
	R: CTGGACCCACTCCTCACTGG	[5,9],	[7]^b^	*CCL3L3*
	P: AGGCCGGCAGGTCTGTGCTGA	[10,17],	[8]^c^	
		[22,23],	[21]^d^	
		[24,25]	[32]^e^	
		[26,27],		
		[28,29],		
		[30,31]		
CCL3L_PP2	F: TTCTGGACCCACTCCTCACT	[12]		*CCL3L1*
	R: CTTCTCCACAGCTTCCTAACCA			*CCL3L3*
	P: FAM-CTGCCGGCCTCTCT			
CCL3L_PP3	F: TGCCTATCTCCGTCTAGAGAGCTT	[13]		*CCL3L1*
	R: AGAAGGAGGCAGCAGGACACT	[33]		*CCL3L2*
	P: FAM-TGACTCCAGGCAAGGG-MGB			*CCL3L3*
CCL3L_PP4	F: GGGTATGACTTCTTGAACCGACAAA	[1]		*CCL3L1*
	R: GGTTCTCTGTTTCTCTATGTGATCCA			*CCL3L3*
	P: 6FAM-CATGAAGAGAGCTAAGAGAAC-MGBNFQ			

**mRNA expression**				
CCL3L_PP5	F: TCTGCAACCAGGTCCTCTCT		[34]^f^	CCL3L1
	R: TTTCTGGACCCACTCCTCAC			CCL3L3
CCL3L_PP6	F:AGCCCTGAACAAAGCATCTG		[35]^g^	CCL3L1
	R:TGAGAGTTCCCCCTGTCCCCT			CCL3L3

**Non-Human**				
	F: CCAGTGCTTAACCTTCCTCC			
CCL3L_PP7	R: TCAGGCACTCAGCTCCAGGT	[11]		
	P: AGGCCGGCAGGTCTGTGCTGACC			

* F = forward primer, R = Reverse primer, P = probe ^a ^SLE; ^b^Rheumatoid arthritis; ^c^Kawasaki disease;^d ^type 1 diabetes mellitus; ^e ^hepatitis C; ^f^Human glioblastoma; ^g^Dendritic cells exposed to contact allergens and irritants

## Results

For the *CCL3L1 *gene, a Pubmed search generated 1059 articles, of which 218 were related to HIV/AIDS. Fifteen of the 218 articles were studies relating to *CCL3L1 *copy numbers; all involved RT-PCR-based assays. Primers and probes used for the studies are summarized in Table 1. Most of these RT-PCR assays were based on the assay described by Towson et al. [17]; a few had modifications. Four studies assessed association of *CCL3L1 *copy numbers with other diseases (Kawasaki disease, SLE, hepatitis C, and rheumatoid arthritis). All of these association studies used RT-PCR assays that were based on the CCL3L_PP1 primer-probe (described in Table 1). Two studies involved use of RT-PCR-based assays to examine mRNA expression of *CCL3L1*.

**Figure 1:**
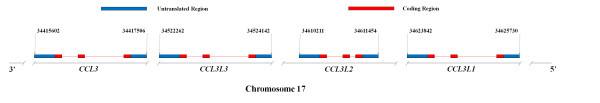
**CCL3L gene cluster**. Annotation, orientation and mapping of the gene cluster *CCL3 (*geneID: 6348), *CCL3L1 *(geneID: 6349), *CCL3L2 *(geneID: 390788) and *CCL3L3 *(geneID: 414062) in the chromosome 17 genomic sequence.

Query coverage of above 70% with 100% maximum identity from the nucleotide blast identified 4 genes, namely *CCL3 (*geneID: 6348), *CCL3L1 *(geneID: 6349), *CCL3L2 *(geneID: 390788), *CCL3L3 *(geneID: 414062), *CCL18 *(geneID: 6362) and *CCL24 *(geneID: 6369). The reference genomic sequences for *CCL3 *- (34415602 - 34417506), *CCL3L1 *- (34623842 - 34625730), *CCL3L2 *- (34610211 - 34611454), *CCL3L3 *- (34522262 - 34524142) were mapped to human chromosome 17 sequence, accession # NC_000017.10 (Figure 1). Of note, *CCL18 *(34391643 - 34398841) in chromosome 17 and *CCL24 *(75441114 - 75443033) also showed high homology (> 50%) sequence. The annotations of exons, introns and untranslated regions of these genes are shown in Figure 1 (see additional file 1 for details). Since *CCL3L2 *is considered a pseudo-gene, locations of these regions were estimated based on the homology this gene shares with *CCL3, CCL3L1 *and *CCL3L3*. As shown in Table 2, the homology in the complete gene region of the four *CCL3L *related genes in chromosome 17q12 was high (55.3 - 98.7%) and even higher in the exonic region (71.9-100%), with identical exonic sequences between *CCL3L1 *and *CCL3L3*.

**Table 2:** Identity matrix (%) between the *CCL3L*-related genes in complete (5'UTR, exons, introns and 3'UTR) genomic sequence (bottom left) and b) exonic regions (top right)

	*CCL3*	*CCL3L1*	*CCL3L2*	*CCL3L3*
***CCL3***	-	0.957	0.713	0.957
***CCL3L1***	0.929	-	0.719	1.000
***CCL3L2***	0.525	0.553	-	0.719
***CCL3L3***	0.921	0.987	0.560	-

The primers and probes from the different studies (labeled CCL3L_PP1-CCL3L_PP7) for both RT-PCR and mRNA expression-based assays showed varying alignments with these four genes (additional file 1), suggesting non-specific amplifications and gene copy number determination. As shown in Table 1, all RT-PCR based assays were specific for both *CCL3L1 *and *CCL3L3*, except CCL3L_PP3, which was specific for *CCL3L1, CCL3L2*, and *CCL3L3 *and CCL3L_PP7 for mRNA expression. The primers/probes used in the study with macaques [11] aligned with the human genomic sequence as well but were nevertheless specific to both *CCL3L1 *and *CCL3L3*.

**Figure 2:**
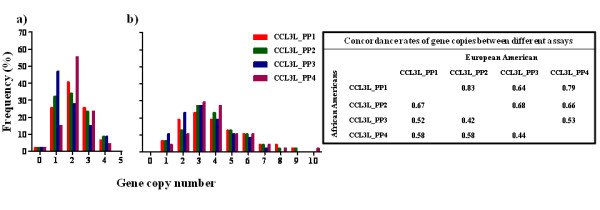
**Gene copy number distribution in Caucasians and African-Americans**. Distribution of CCL3L-related gene copy number by four RT-PCR assays: CCL3L_PP1, CCL3L_PP2, CCL3L_PP3, and CCL3L_PP4 in a) 47 Caucasians and b) 48 African-Americans. The concordance rates (same gene copy calls) between different assays are shown in the table.

Figure 2 illustrates the distribution of CCL3L-related gene copy number based on the four different assays in a) Caucasians and b) African-Americans. Based on the sequence alignment (additional file 1), while CCL3_PP1, CCL3L_PP2, and CCL3L_PP4 are specific to *CCL3L1 *and *CCL3L2*, the assay CCL3_PP3 is specific to all three genes; *CCL3L1*, *CCL3L2 *and *CCL3L3*. The median gene copy was 2 for all assays in European Americans; 3 for CCL3L_PP3 and 4 for CCL3L_P1, CCL3L_P2, and CCL3L_P4 in African Americans and the means were 2.08, 2.04, 1.84 and 2.13 in European Americans and 3.93, 3.94, 3.3 and 4.04 in African Americans for CCL3_PP1, CCL3L_PP2, CCL3L_PP3 and CCL3L_PP4, respectively. Interestingly, CCL3L_PP1 had the lowest mean in both ethnic groups and CCL3L_PP4 had the highest mean and median in both ethnic groups. The concordance rates of gene copy calls were 83%, 64% and 79% between CCL3L_PP1 and CCL3L1_PP2, CCL3L1_P3, and CCL3L1_P4 assays, respectively in European Americans and 67%, 52%, and 58%, respectively in African Americans; 68% and 66% between CCL3_PP2 and CCL3_PP3 and CCL3_PP4, respectively in European Americans and 42% and 58%, respectively in African Americans and 53% between CCL3_PP3 and CCL3_PP4 in European Americans and 44% in African Americans. Overall, the concordance rate seems to be relatively higher in European Americans than African Americans where the gene copy calls are higher with wider distributions.

## Discussion

The non-specificity of different assays for *CCL3L1 *gene copy number raises the question of the gene specificity reported in earlier studies. Until the genetic architecture of this complex region is understood, it will be difficult to evaluate the genes individually and determine if there is an association between the copy numbers of all the genes in the cluster or if the association is specific to the copy number of only one gene [18]. Further, there might be situations in which a higher gene copy of one gene is detrimental and a higher gene copy of another is beneficial. Based on existing assays, the distribution of copy numbers of *CCL3L1/CCL3L3 *differs in different ethnic groups, (e.g., median copies of two, four, and six in Caucasians, Asians, and Africans, respectively) [12,13]. In our own results, while this seems to be the case at the population level, there seems to be inconsistencies between the different assays at the individual level, as shown specifically by the low concordance rates (Figure 2). Overall, CCL3L_PP4 has a higher mean than other assays both in African American and European American could possibly measure all three genes as previously described [1], but the sequence alignment shows that the assays are based specific to CCL3L1 and CCL3L3 and not to the truncated CCL3L2. Nevertheless, CCL3L_PP4 is not consistently larger across samples. On the contrary, CCL3L_PP4 which binds to all three genes has actually the lowest mean copy number. This clearly suggests non-specificity of the assays regardless of the sequence alignment.

If *CCL3L1 *and *CCL3L3 *are both diploid in nature then an individual should possess 4 copies of these genes based on the assays specific to two of these genes (Table 1: CCL3L_PP1, CCL3L_PP2, CCL3L_PP4, and CCL3L_PP5), so it is puzzling why there are several individuals with 1 or 2 copies, especially among Caucasians as shown in Figure 2. Do they lack one of these genes completely or do they have one copy of each or do they have different copy number of each gene? Any such discrepancy may confound the association from the biological function of the expressed proteins rather than the copies of the sequence in the genome. Thus, further investigation is needed to clarify the distribution for the sub-fractions of these genes and to understand how immunity with respect to the affect of each of these genes has evolved in different populations, including non-human primates, who tend to have higher copy numbers. Theoretically any assay performed to determine total *CCL3L1 *and *CCL3L3 *copies (although does not distinguish the dose of *CCL3L1 *and *CCL3L3 *in each individual) should reveal the same results and thus there should be no effect in the association. However, as we see from our results, the concordance rates are low. Without a gold-standard it is not possible to reliably assess which assay is better, but any misclassifications could lead to incorrect associations. Additionally, there are several other issues such as dye chemistry, reaction specificity/conditions and DNA concentrations that might also affect the assays [19]. Even with exact copy numbers, while there may not be differences at the sequence level, there may be differences at the expression levels and therefore may confound the overall association at the protein level rather than the nucleotide sequence level. For instance, the affinity of CCL3L1 is strongest for CCR5 and may be important to know how many copies of *CCL3L1 *an individual has versus how many of total *CCL3L1 *and *CCL3L3*. It remains to be shown how these two genes are differentially expressed and also how an expression of one gene might be affected by differential copy numbers of the others. They could enhance the affinity, reduce the affinity, or have no effect. However, while the expression and protein levels are important, the structural variants of these genes at the sequence level (copy numbers) needs to be understood and assayed properly to determine which ones are functional and what their levels are.

Since the extent of the non-specificity of the current RT-PCR based assays has not been well defined, the comparison to delineate the homologous regions provides basic information for assay development. The present data show that other genes, such as *CCL18 *and *CCL24*, which may not be in the gene cluster, also have overlapping regions (30-34% in CCL18 and 27-44% in CCL24 with genes in CCL3L cluster). At present, different bio-informatics tools are needed to examine the sequences and to understand their complexities. While our inferences of the RT-PCR based assays are based on a single reference genome assembly, all previous assays were likely based on the reference sequence as well and thus our approach provides a less conservative specificity since polymorphisms between and within genes in a population could further confound the specificity of the primers/probes. For example, although the exonic sequences between *CCL3L1 *and *CCL3L3 *are identical, there is at least one SNP in the UTR and two in the introns of *CCL3L1 *and one in the intron of *CCL3L3 *(additional file 1) that are uniquely polymorphic (based on the reference genome) and can be utilized to develop assays. However, it is yet to be determined if these are different within genes or between genes to make a more reliable assay. Additionally, variants between and within copies of the CCL3L-related genes might influence the function of these genes. For example, SNPs in *CCL3 *and *CCL3L1 *genes determine their production [20]. Thus, SNPs and copy numbers are important in examining the production and expression of these gene levels. Both should be assayed appropriately.

Recently, as an alternative to RT-PCR, a method for *CCL3L1 *copy number determination based on a paralogue ratio test (PRT) has been developed [21]. However, the primers are non-specific and align with both *CCL3L1 *and *CCL3L3*. With the current methodologies for determining gene copy numbers, a main assumption is that the derived gene copy number based on specific probes represents the whole gene. This may not always be the case, since only parts of the gene might be duplicated; this may be missed if the probe is not specific for this segment or may provide a false count when other segments of the gene are not amplified, especially the functional segments. In some cases, there might be a complete gene copy, but subtle differences may be present at the sequence level. The orientation of the gene might be opposite so that expression would not be the same, or there might be differences in single-nucleotide polymorphisms (SNPs) between copies. To account for these variables, complete sequence data of all copies could be required. In summary, we report homology at the nucleotide sequence level between the different *CCL3L*-related gene clusters and primers/probes for the RT-PCR based assays. The currently used assays for gene copies of *CCL3L1 *are evidently non-specific and thus could overestimate the copy numbers. Based on the overlapping and non-specific sequences between these genes, current gene copy assays, such as gene specific RT-PCR, pyrosequencing, paralogue ratio tests (PRT), multiplex amplifiable probe hybridization (MAPH) or multiplex ligation-dependent probe amplification (MPLA), could be fine-tuned with broad-range nested PCR methods to avoid redundant sequences and other new assays developed. Special precautions, however, are needed to avoid the homologous sequences. Non-specificity of the laboratory methods for CNVs should not be overlooked as we develop different analytical methods to account for heterogeneity in association results.

## Competing interests

The authors declare that they have no competing interests.

## Authors' contributions

SS conceived ideas to examine assays at the sequence level, re-examined the bioinformatics and drafted the manuscript. MN conducted the literature search, performed the bioinformatics and the laboratory assays. JCE assisted with the supervision of the assays, analysis and interpretation. All three authors read and approved the final manuscript.

## Supplementary Material

Additional file 1 **Multiple primer-probe alignment with chemokine genes**. Multiple alignments of the primer-probe pairs used for RT-PCR based assays in different studies with genomic sequences for four chemokine genes: *CCL3, CCL3L1, CCL3L2 and CCL3L3*. The sequences of the genes were obtained as follows: *CCL3 *- (34415602 - 34417506), *CCL3L1 *- (34623842 - 34625730), *CCL3L2 *- (34610211 - 34611454), and *CCL3L3 *- (34522262 - 34524142) from the genomic sequence of chromosome 17 sequence (Accession Number NC_000017.10). For each gene, untranslated region sequences are highlighted in blue, exons in red, and introns in green. Missing sequences are marked as "-"to optimize gene alignments. (Differences in the gene sequences are marked as follows: "*" = *CCL3L*, "‡" = *CCL3L1*, "Δ" = *CCL3L2*, and "†" = *CCL3L3*) Click here for file
