# Supplementary material for: Variations in CCL3L gene cluster sequence and non-specific gene copy numbers
Source: BMC Res Notes. 2010 Mar 16;3:74. doi: 10.1186/1756-0500-3-74 (PMC2851716; doi:10.1186/1756-0500-3-74)
Supplement: Additional file 1 — Multiple primer-probe alignment with chemokine genes. Multiple alignments of the primer-probe pairs used for RT-PCR based assays in different studies with genomic sequences for four chemokine genes: CCL3, CCL3L1, CCL3L2 and CCL3L3. The sequences of the genes were obtained as follows: CCL3 - (34415602 - 34417506), CCL3L1 - (34623842 - 34625730), CCL3L2 - (34610211 - 34611454), and CCL3L3 - (34522262 - 34524142) from the genomic sequence of chromosome 17 sequence (Accession Number NC_000017.10). For each gene, untranslated region sequences are highlighted in blue, exons in red, and introns in green. Missing sequences are marked as "-"to optimize gene alignments. (Differences in the gene sequences are marked as follows: "*" = CCL3L, "‡" = CCL3L1, "Δ" = CCL3L2, and "†" = CCL3L3) [file 1756-0500-3-74-S1.DOC]

**Additional File 1.**

**COLOR = Untranslated Region COLOR = Exon COLOR = Intron**

**CCL3**  **------------------------------------------------------------------------------------------**

**CCL3L1**  **------------------------------------------------------------------------------------------**

**CCL3L2**  **------------CC---AATTTCCCTCCATTTCACCTCTTCC---TAATCTTTGCCTATGATTCCTCTTAACCAGTGATTTTGATTTGCC**

**CCL3L3**  **------------------------------------------------------------------------------------------**

***CCL3L_PP7_F***

**AGCCCTGAACAAAAG**

**CCL3**  **----------------------------TTTACCTTTTAAAAGAGCATCTTTATTATTTCCCCAGGCCGATCACAGCCCTGAACAAAAG**

**CCL3L1**  **----------------------------TTTACCGTTTAAAAGAACATCTTTATTATTTCCCCAGGCCGATCACAGCCCTGAACAAAAG**

**CCL3L2**  **AGAAAAACAAAACCAAACTCAATACTGGTTTACCGTTTAAAAGAACATCTTTATTATTTCCCCAGGCCGATCACAGCCCTGAACAAAAG**

**CCL3L3**  **----------------------TACTGGTTTACCGTTTAAAAGAACATCTTTATTATTTCCCCAGGCCGATCACAGCCCTGAACAAAAG**

* *

**CATCTG**

**CCL3**  **CATCCGATACACATTTGTCAGTCTGGTGGCTTTGGTGCCATGACTGCCTACACAGGCTGATGACAGCCACTCGGTTGTCACCAG**

**CCL3L1**  **CATCTGATACACATTTGTCAGTCTGGTGGCTTTGGTGCCATGACTGCCTACACAGGCCGATGACAGCCACTCGGTTGTCACCAG**

**CCL3L2**  **CATCTGATACACATTTGTCAGTCTGGTGGCTTTGGTGCCATGACTGCCTACACAGGCCGATGACAGCCACTCGGTTGTCACCAG**

**CCL3L3**  **CATCTGATACACATTTGTCAGTCTGGTGGCTTTGGTGCCATGACTGCCTACACAGGCCGATGACAGCCACTCGGTTGTCACCAG**

* *

***CCL3L_PP7_R***

**AGGGGACAGGGGGAACTCTCA**

**CCL3**  **ACGCGGTGTGAGGGAAGGGGGAGGGGACAGGGG-AACTCTCAGAGCAAACAATCACAAACACACTGTGAAATCGAAAATAA**

**CCL3L1**  **ACACACTGTGAGGGAAGGTGGAGGGGACAGGGGGAACTCTCAGAGCAAACAATCACAAACACACTGTGAAATCAAAAATAA**

**CCL3L2**  **ACACACTGTGAGGGAAGGTGGAGGGGACAGGGGGAACTCTCAGAGCAAACAATCACAAACACACTGTGAAATCAAAAATAA**

**CCL3L3**  **ACACACTGTGAGGGAAGGTGGAGGGGACAGGGGGAACTCTCAGAGCAAACAATCACAAACACACTGTGAAATCAAAAATAA**

* ** * *

**CCL3**  **ATTACAAAAACTAAATAGTATAAATAAATTAAAATTTAAGTTAAGAAGAGTCCCACAGTGTGGCTGTTTGGCAACAACCAGTCCATAGA**

**CCL3L1**  **ATTATAAAAACTAAATAGTATAAATAAATTAAAATTTAAGTTAAGAAGAGTCCCACAGTGTGGCTGTTTGGCAATAACCAGTCCATAGA**

**CCL3L2**  **ATTATAAAAACTAAATAGTATAAATAAATTAAAATTTAAGTTAAGAAGAGTCCCACAGTGTGGCTGTTTGGCAATAACCAGTCCATAGA**

**CCL3L3**  **ATTATAAAAACTAAATAGTATAAATAAATTAAAATTTAAGTTAAGAAGAGTCCCACAGTGTGGCTGTTTGGCAATAACCAGTCCATAGA**

* *

**CCL3**  **AGAGGTAGCTGTGGAGGTCACACGCATGTTCCCAAGGCTCAGGCTCCTGCTCCTCCCCACTGGGCCCACCGAGGTCGCTGGGCCT**

**CCL3L1**  **AGAGGTAGCTGTAGAGGTCACACGCATGTTCCCAAGGCTCAGGCTCCTGCTCCTCCCCACTGGGCCCACTGAGGTCGCTGGGCCT**

**CCL3L2**  **AGAGGTAGCTGTGGAGGTCACACGCATGTTCCCAAGGCTCAGGCTCCTGCTCCTCCGCACTGGGCCCACTGAGGTCGCTGGGCCT**

**CCL3L3**  **AGAGGTAGCTGTGGAGGTCACACGCATGTTCCCAAGGCTCAGGCTCCTGCTCCTCCCCACTGGGCCCACTGAGGTCGCTGGGCCT**

‡ ∆ *

***CCL3L_PP2_F***

**TTCTGGACCCACTCCTCACT**

***CCL3L_PP1_R*** ***CCL3L_PP1_P***

**CTGGACCCACTCCTCACTGG TCAGCACAG**

***CCL3L_PP4_R*** ***CCL3L_PP4_P***

**TCAGGCACTCAGCTCCAGGT** **GGTCAGCACAG**

***CCL3L_PP6_R***

**TTTCTGGACCCACTCCTCAC**

**CCL3**  **CGAAGCTTCTGGACCCCTCAGGCACTCAGCTCCAGGTCGCTGACATATTTCTGGACCCACTCCTCACTGGGGTCAGCACAG**

**CCL3L1**  **CGAAGCTTCTGGACCCCTCAGGCACTCAGCTCCAGGTCACTGACGTATTTCTGGACCCACTCCTCACTGGGGTCAGCACAG**

**CCL3L2**  **CGAAGCTTCTGGACCCCTCAGGCACTCGGCTCCAGGTCACTGACGTATTTCTGGACCCACTCCTCACTGGGGTCAGCACAG**

**CCL3L3**  **CGAAGCTTCTGGACCCCTCAGGCACTCAGCTCCAGGTCACTGACGTATTTCTGGACCCACTCCTCACTGGGGTCAGCACAG**

∆ * *

***CCL3L_PP2_P CCL3L_PP2_R***

**CTGCCGGCTTCTCTTGGTTAGGAAGCTGTGGAGAAG**

***CCL3L_PP1_F***

**ACCTGCCGGCCT TCTTGGTTAGGAAGCTGTGGAGA**

***CCL3L_PP4_F***

**ACCTGCCGGCCT** **GGAGGAAG-GTTAAGCACTGG**

**CCL3**  **ACCTGCCGGCTTCGCTTGGTTAGGAAGCTGTGGAGAAGGGAGGAAGAGTTAAGCACTGGGGAATCCAGCAGGGGAATCCTGGGCCCA**

**CCL3L1**  **ACCTGCCGGCCTCTCTTGGTTAGGAAGCTGTGGAGAAGGGAGGAAGAGTTAAGCACTGGGGAATCCAGCCGGGGAATCCTGGGCCCA**

**CCL3L2**  **ACCTGCCGGCCTCTCTTGGTTAGGAGGCTGTGGAGAAGGGAGGAAGAGTTAAGCACTGGGGAATCCAGCCGGGGAATCCTGGGCCCA**

**CCL3L3**  **ACCTGCCGGCCTCTCTTGGTTAGGAAGCTGTGGAGAAGGGAGGAAGAGTTAAGCACTGGGGAATCCAGCCGGGGAATCCTGGGCCCA**

* * ∆ *

***CCL3L_PP3_F***

**TGCCTATCTCCGTCTAGAGAGCTT**

**CCL3**  **CCATGGCCCCACCATTCTGCTCTCTGTCCTGGGCAGCTCAGGGCTTGCTCCTCTTTCAGGGGCCCCCTGCCTATCTCTGTCTAGAGAGCTT**

**CCL3L1**  **CCATGGCCCTGACATCCTGCTCTCTGTCCTGGGCAGCTCAAGGCCTGCTCCTCTCTCAGGGGCCCCCTGCCTATCTCCGTCTAGAGAGCTT**

**CCL3L2**  **CCATGGCCCTGACATCCTGCTCTCTGTCCTGGGCAGCTCAAGGCCTGCTCCTCTCTCAGGGGCCCCCTGCCTATCTCCGTCTAGAGAGCTT**

**CCL3L3**  **CCATGGCCCTGACATCCTGCTCTCTGTCCTGGGCAGCTCAAGGCCTGCTCCTCTCTCAGGGGCCCCCTGCCTATCTCCGTCTAGAGAGCTT**

*** * * * * *

***CCL3L_PP3_P CCL3L_PP3_R***

**TGACTCCAGGCAAGGG AGTGTCCTGCTGCCTCCTTCT**

**CCL3**  **CTCTCAGTGACTC-AGTAGGGGTGGCCCTCAGAGTGTCCCGCTGCCTCCTTCTTCCTGTCCCTTTCCTCTGGGCTGGGGCAGCCCTTC**

**CCL3L1**  **CTCTCAGTGACTCCAGGCAAGGGGGCCCTCAGAGTGTCCTGCTGCCTCCTTCTTCCTGTTCCTTTCCTCTGGCCTGGGGCAGCCCTTC**

**CCL3L2**  **CTCTCAGTGACTCCAGGCAAGGGGGCCCTCAGAGTGTCCTGCTGCCTCCTTCTTCCTGTTCCTTTCCTCTGGCCTGGGGCAGCCCTTC**

**CCL3L3**  **CTCTCAGTGACTCCAGGCAAGGGGGCCCTCAGAGTGTCCTGCTGCCTCCTTCTTCCTGTCCCTTTCCTCTGGCCTGGGGCAGCCCTTC**

* **** * * ‡ *

∆

**CCL3**  **CTGACTCTGTAACACATGCCTCACTCCAGCTCCAAGTCAGGTCACACCTCGGAGCCCTGCGTCCTGTATCCCCGATAGGCTCCTGA**

**CCL3L1**  **CTGACTCTGTAACACCCACCTCACTCCAGCCCCAAGTCAGGTCACACCTCAGTGCCCTGCGTCCTGTATCCCCGATAGGCTCCTGA**

**CCL3L2**  **CTGACTCTGTAACACCCACCTCACTCCAGCCCCAAGTCAGGTCACACCTCAGTGCCCTGCGTCCTGTATCCCCGATAGGCTCCTGA**

**CCL3L3**  **CTGACTCTGTAACACCCACCTCACTCCAGCCCCAAGTCAGGTCACACCTCAGTGCCCTGCGTCCTGTATCCCCGATAGGCTCCTGA**

*** * *

**CCL3**  **AGGCTGGGCCTTTCCAGGATGGCCTTCTGGCCTGTCTCTGCCCCAACCCTGACCCTCCCTACCTCCATAGAGGTGAGCAGGAAGA**

**CCL3L1**  **AGGCTGGGCCTTTCCAGGATGGCCTTCTGGCCTGTTTCTGCCCCCACCCTGACACTCCCTACCTCCCTAGAGGTGAGCAGGAAGA**

**CCL3L2**  **AGGCTGGGCCTTTCCAGGATGGCCTTCTGGCCTGTTTCTGCCCCCACCCTGACACTCCCTACCTCCCTAGAGGTGAGCAGGAAGA**

**CCL3L3**  **AGGCTGGGCCTTTCCAGGATGGCCTTCTGGCCTGTTTCTGCCCCCACCCTGACACTCCCTACCTCCCTAGAGGTGAGCAGGAAGA**

* * * *

**CCL3**  **CTGGCACTTACATGACACCGGGCTTGGAGCACTGGCTGCTCGTCTCAAAGTAGTCAGCTATGAAATTCTGTGGAATCTGCCGGGAGGTGT**

**CCL3L1**  **CTGGCACTTACATGACACTGGGCTTGGAGCACTGGCTGCTCGTCTCAAAGTAGTCAGCTATGAAATTCTGTGGAATCTGTCGGGAGGTGT**

**CCL3L2**  **CTGGCACTTACATGACACTGGGCTTGGAGCACTGGCTGCTCGTCTCAAAGTAGTCAGCTATGAAATTCTGTGGAATCTGCCGGGAGGTGT**

**CCL3L3**  **CTGGCACTTACATGACACTGGGCTTGGAGCACTGGCTGCTCGTCTCAAAGTAGTCAGCTATGAAATTCTGTGGAATCTGTCGGGAGGTGT**

* ‡

†

**CCL3**  **AGCTGAAGCAGCAGGCGGTCGGCGTGTCAGCAGCAACTGTGGAGAAAGGAAGAGAATAAGCCCGAGTCACAGCTCAGAAGAAAAGG**

**CCL3L1**  **AGCTGAAGCAGCAGGCGGTCGGCGTGTCAGCAGCAACTGCGGAGAAAGGA-GAGAATAAGCCCGAGTCACAGCTCAGAAGAAAAGG**

**CCL3L2**  **AGCTGAAGCAGCAGGCGGTCGGCGTGTCAGCAGCAACTGCGGAGAAAGGA-GAGAATAAGCCCAACTCT--GTATGTGTGCATATG**

**CCL3L3**  **AGCTGAAGCAGCAGGCGGTCGGCGTGTCAGCAGCAACTGCGGAGAAAGGA-GAGAATAAGCCCGAGTCACAGCTCAGAAGAAAAGG**

* * ∆ ∆ ∆∆∆∆ ∆∆∆∆∆∆∆ ∆ ∆∆∆∆

**CCL3**  **CCAGGCAGCTTCTGATCCCCGAGCAGTTGAGGAAGGCAGGCTTGCTCAGACCAAGTGACTGGAAGGCATTTGGGCATTTTTGCTGAGA**

**CCL3L1**  **CCAGGCAGCTTCTGATCCCTGAGTGGTTGAGGAGGGCAGGCTTGCTCAGACCAAGTGACTGCAAGGCATTTGGGGGGTTTTGCAGAGA**

**CCL3L2**  **TTTGTGTGTGTGTG-TCCTACAGGTGTGAGCGAGTGGATGCTTCC----ACTACGTGT------------------------------**

**CCL3L3**  **CCAGGCAGCTTCTGATCCCTGAGTGGTTGAGGAGGGCAGGCTTGCTCAGACCAAGTGACTGCAAGGCATTTGGGGGGTTTTGCAGAGA**

∆∆∆ ∆∆∆ ∆∆∆∆ ∆ ∆∆∆ ∆∆ ∆∆∆∆ ∆∆ ∆ ∆ ∆∆ ∆ ∆ ∆ * *** *

* *

***CCL3L_PP5_R***

**GGTTCTCTGTTTCTCTATGTGATCCA**

**CCL3**  **AATGTCTCTTTGTTTCTGTCTGTATCCTTCTTTCTCCTTGACTCTTCATAGTGGGTTCTCTGTTTCTCTGTGTGATCCAGATACCTGAAC**

**CCL3L1**  **AATGTCTCTTTGTTTCTGTCTATATTCCTCTTTCCCCTTGACTCTTCATAGTGGGTTCTCTGTTTCTCTATGTGATCCAGATACCTGAAT**

**CCL3L2**  **------------------------------------------------------------------------------------------**

**CCL3L3**  **AATGTCTCTTTGTTTCTGTCTATATTCCTCTTTCCCCTTGACTCTTCATAGTGGGTTCTCTGTTTCTCTATGTGATCCAGATACCTGAAT**

* * * * * *

***CCL3L_PP5_P CCL3L-PP5_F***

**GTTCTCTTA-----GCTCTCTTCATG TTTGTCTGGTTCAAGAAGTCATACCC**

**CCL3**  **GGACTGTTCTCTTATCTCAGTTCTCTTCAGGGAATTTTGTCTGGTTCAAGAAGTCATACCCCAACCCAAGAGAAGCCTTGGACATCTCTC**

**CCL3L1**  **GGACTGTTCTCTTA-----GCTCTCTTCATGGAATTTTGTC-GGTTCAAGAAGTCATACCCCAGCCCAAGAGAAGCCCTGGACATCTCTC**

**CCL3L2**  **------------------------------------------------------------------------------------------**

**CCL3L3**  **GGACTGTTCTCTTA-----GCTCTCTTCATGGAATTTTGTC-GGTTCAAGAAGTCATACCCCAGCCCAAGAGAAGCCCTGGACATCTCTC**

***** * * * * *

**CCL3**  **ATAAGACATCCAAGGGACAGAGCTCCTGGGAGACCTAGAGTGAGCTGGAGAGTGAACAACATACCCCACTGGGAAGTAAGCAGCCCTGGA**

**CCL3L1**  **ATAAGACATCCAAGGGACAGGGCTCCTGGGAGACCTAGGGTGAGCTGGAGAGTGAACAACAGACCCCACTGGGAAGTAAGCAGCCCTGGA**

**CCL3L2**  **------------------------------------------------------------------------------------------**

**CCL3L3**  **ATAAGACATCCAAGGGACAGGGCTCCTGGGAGACCTAGGGTGAGCTGGAGAGTGAAGAACAGACCCCACTGGGAAGTAAGCAGCCCTGGA**

* * † *

**CCL3**  **TTCTGCCTCTTGCTAACTGATTCGTTTCGAACCCTGTTTTTCTATCTGTAAAAGGGACT-GTAACTCCCCTGCCCCTGCCTAGATTCTCA**

**CCL3L1**  **TTCTGCCTCTTGCAAACTGATTCGTTTTGAACCCTGTTTTTCTATCTGTACAAGGGACT-GTAACTCCCCTGCCCCTGCCTAGATTCTCA**

**CCL3L2**  **------------------------------------------------------------------------------------------**

**CCL3L3**  **TTCTGCCTCTTGCAAACTGATTCGTTTTGAACCCTGTTTTTCTATCTGTACAAGGGACT-GTAACTCCCCTGCCCCTGCCTAGATTCTCA**

* * *

**CCL3**  **TACCTGGAGACTAGG-GGGCTAAGACCCCTTCTAGAGATAAAAATAAAAGTCTTAAAGAGAAAGACCAAGATGTTTGGCAGCCCTTTAAG**

**CCL3L1**  **TACCTGGAGACTAGGAGGGCTAAGACCCCTTCTAGAGATAAAAATAAAAGTTGTGAAGAAAAAGACCAAGGTGTTTGGCAGCGCTTTAAG**

**CCL3L2**  **------------------------------------------------------------------------------------------**

**CCL3L3**  **TACCTGGAGACTAGGAGGGCTAAGACCCCTTCTAGAGATAAAAATAAAAGTTGTGAAGAAAAAGACCAAGGTGTTTGGCAGCGCTTTAAG**

* ** * * * *

**CCL3**  **AAGTTC--TCTTTTCTCTTGGGGGCTTTTAGGCCACAAGAAAAGATTGATGTGGTCTAACCAT--GGCCAGAGAGTGGTGATACC--CAC**

**CCL3L1**  **AACTTCCTTCTTTTCTCTTCGGGGCTCTCAGGCCGCAAAAAAAGACTGATGTGGTCTAACCAT--GGCCAGAGAGTGGTGATACC--CAC**

**CCL3L2**  **------------------------------------------------------------------------------------------**

**CCL3L3**  **AACTTCCTTCTTTTCTCTTCGGGGCTCTCAGGCCACAAAAAAAGACTGATGTGGTCTAACCAT--GGCCAGAGAGTGGTGATACC--CAC**

* ** * * * ‡ * *

***CCL3L-PP6_R***

**AGAGAGGACCTGGTTGCAGA**

**CCL3**  **AACGA-AACTCAGACTCACGTGATGCAGAGAA---CTGGTTGCAGAGAGCCATGGTGCAGAGGAGGACAGCAAGGGCAGCAGTGGAGACC**

**CCL3L1**  **AACAACAACATGGACTCACGTGGTGCAGAGAGGACCTGGTTGCAGAGAGCCATGGTGCAGAGGAGGACGGCAAGGGCAGCAGTGGAGACC**

**CCL3L2**  **------------------------------------------------------------------------------------------**

**CCL3L3**  **AACAACAACATGGACTCACGTGGTGCAGAGAGGACCTGGTTGCAGAGAGCCATGGTGCAGAGGAGGACGGCAAGGGCAGCAGTGGAGACC**

* * *** * **** *

**CCL3**  **TGCATGATTCTGAGCAGGTGACGGAATGTGGGCTCGAGTGTCAGCAGAGCCAAGAAAGGACTGACCACTGTCTGCTGCCCGTGTC-CTTC**

**CCL3L1**  **TGCATGATTGGGAGCAGGTGATGGAATGTGGGCTCGAGTGTCAGCAGAGCCAAGAAGGGACTGACTACTCTTTGCTGCCTGCGTC-CTTC**

**CCL3L2**  **------------------------------------------------------------------------------------------**

**CCL3L3**  **TGCATGATTGGGAGCAGGTGATGGAATGTGGGCTCGAGTGTCAGCAGAGCCAAGAAGGGACTGACTACTCTTTGC---------------**

** * * * * * * *

**CCL3**  **TGAAGTCTGAAACCAGCT------------------------------------------------------------------------**

**CCL3L1**  **------------------------------------------------------------------------------------------**

**CCL3L2**  **------------------------------------------------------------------------------------------**

**CCL3L3**  **------------------------------------------------------------------------------------------**
